# Parkinson’s Disease: A Nanotheranostic Approach Targeting Alpha-Synuclein Aggregation

**DOI:** 10.3389/fcell.2021.707441

**Published:** 2021-08-20

**Authors:** Fong LaiGuan Zoey, Mathangi Palanivel, Parasuraman Padmanabhan, Balázs Gulyás

**Affiliations:** ^1^Lee Kong Chian School of Medicine, Nanyang Technological University, Singapore, Singapore; ^2^Imaging Probe Development Platform, Nanyang Technological University, Singapore, Singapore; ^3^Cognitive Neuroimaging Centre, Nanyang Technological University, Singapore, Singapore

**Keywords:** alpha-synuclein, Parkinson’s disease, magnetic resonance imaging, nanotechnology, nanoparticle, diagnostic, therapeutic, nanotheranostic

## Abstract

Parkinson’s disease (PD) is one of the most common neurodegenerative disorders that is implicated in aging populations. As numerous developed nations are experiencing progressively aging populations today, there is a heightened propensity for the occurrence of PD cases. Alpha-synuclein (α-syn) aggregation has been considered to be the pivotal mechanism leading to PD pathogenesis. Thus, early diagnostic and therapeutic strategies targeting the misfolded α-syn protein can potentially improve the prognosis of PD. With rapid advancements in nanotechnology in the last decade, effective solutions to various neurodegenerative and oncological diseases have been suggested. This review will explore the current innovations in nanotechnology that target the α-syn aggregation pathway, and reinstate the promise they hold as effective early diagnostic and therapeutic solutions to PD.

## Introduction

Parkinson’s disease (PD) is the second most widespread neurodegenerative disorder of aging (Fields et al., 2019). The key pathological features of PD include the Lewy pathology, dopaminergic neuronal death and a subsequent dopamine depletion in the nigrostriatal and mesolimbic system, which result in movement abnormalities, cognitive and psychiatric manifestations (Ibarretxe-Bilbao et al., 2011). The cardinal motor symptoms of PD comprise resting tremors, rigidity, bradykinesia, and postural instability. Furthermore, frequently implicated cognitive and psychiatric manifestations include dementia and depression, with some occurrence of hallucinations, irritability, apathy, and anxiety (Stacy, 2011). PD may result in severe disability, which leads to massive social and economic burden to the population. As numerous developed nations are experiencing progressive aging populations today, there is a widespread increase in and anticipated heightened propensity for the occurrence of PD cases in the future. Despite this phenomenon, the majority of PD cases remain idiopathic, with some attributed to genetics and certain toxins (Lill and Klein, 2017).

A defining neuropathological feature of PD is the accumulation of Lewy bodies in different brain regions, which are primarily composed of misfolded and aggregated alpha-synuclein (α-syn) (Spillantini et al., 1997). There is increasing evidence that underscores the pivotal role of α-syn in PD pathogenesis. α-syn is a protein composed of 140 amino acids typically enriched in the presynaptic vesicular compartment, which is known to promote SNARE complex formation, a phenomenon that is essential for the regulation of vesicle dynamics and neurotransmitter transmission (Burré et al., 2011). Recent studies have also proposed that α-syn modulates neuronal DNA repair, microtubule-polymerizing activity, and the maintenance of cellular membrane bilayer (Schaser et al., 2019). The homeostasis of α-syn in the brain is maintained by optimal rates of protein synthesis, aggregation, and clearance. An imbalance in these mechanisms might cause an accumulation of α-syn which will favor aggregate formation. An accumulation of α-syn aggregates can lead to a disruption in synaptic regulation, impairment of neuronal signaling and eventual neuronal death (Volpicelli-Daley et al., 2011).

**FIGURE 1:**
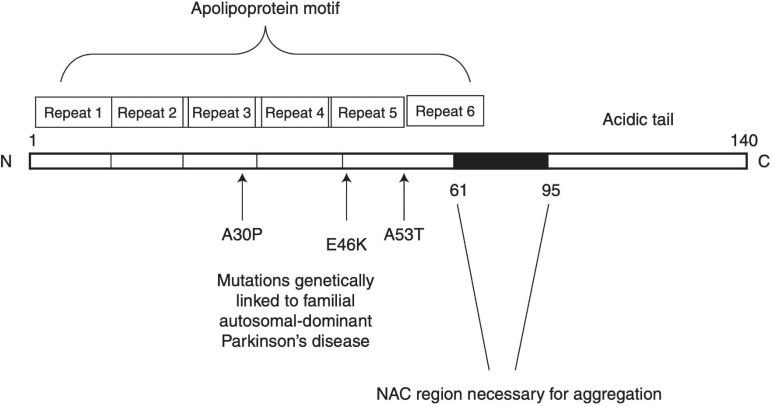
Schematic structure of α-synuclein. Reprinted from Stefanis (2012), with permission from Cold Spring Harbor Laboratory Press.

The incidence of PD is primarily idiopathic, with less than 10% of cases being inherited. In some cases of inherited PD, mutations of the *SNCA* gene cause an increase in α-syn expression and neurotoxicity (Chartier-Harlin et al., 2004; Simón-Sánchez et al., 2009). The α-syn oligomerization and fibril growth processes have been proposed to partake in a pivotal role in the pathogenesis of PD. α-syn is composed of three distinct regions: (1) an amino terminus (residues 1–60) that confers α-syn the ability to form α-helical structures upon membrane binding. This is where all known SNCA familial PD mutations occur. (2) A central hydrophobic region (61–95) known as non-amyloid-β component (NAC) that offers β-sheet potential, which is essential for α-syn aggregation. (3) A highly negatively charged carboxy terminus that is prone to phosphorylation at tyrosine 125 and serine 129 (McFarland et al., 2008; Kumari et al., 2021). A schematic structure of α-syn is depicted in Figure 1. Owing to these properties, α-syn under normal conditions is natively unfolded in aqueous solution, but forms α-helical structures when binding to negatively charged lipids, such as phospholipids on cellular membranes, or β-sheet-rich structures (Stefanis, 2012). When overexpressed or misfolded due to either genetic mutation or protein modification, α-syn fibrils can accumulate and aggregate into soluble oligomers and protofibrils, which may eventually stabilize into insoluble amyloid fibrils structures. Soluble oligomers and protofibrils, formed during the early phases of α-syn fibrillation, have been shown in various studies to be particularly toxic as compared to the insoluble amyloid-like fibrils that ultimately form Lewy bodies (Chen et al., 2007; Danzer et al., 2007; Winner et al., 2011). It was suggested that α-syn interaction with lipids may play a role in its aberrant effects. Sequestration of the fatty acid arachidonic acid by α-syn away from the SNARE complex has been suggested to underlie its inhibitory effect on neuronal transmission (Darios et al., 2010). Polyunsaturated fatty acids (PUFAs) have been shown to enhance oligomerization and neurotoxicity of α-syn (Assayag et al., 2007). Since dopamine and its metabolites can inhibit the conversion of protofibrils to mature fibrils *in vitro* and *in vivo* through the formation of dopamine/α-syn adducts, soluble oligomers were suggested to play a key role in the preferential vulnerability of dopaminergic neurons to the neurotoxic effects of αSyn (Conway et al., 2001).

**FIGURE 2:**
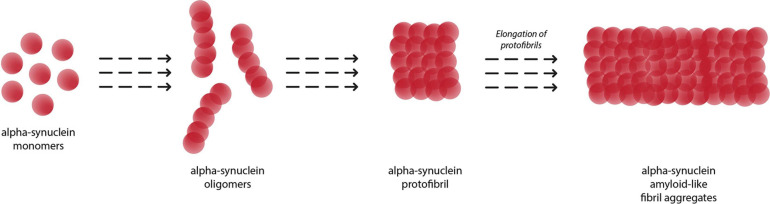
Aggregation of alpha-synuclein monomers into eventual insoluble fibrils, with oligomer and protofibril formation as intermediaries.

Figure 2 depicts the conversion of α-syn monomers into oligomers, protofibrils and eventually insoluble fibril aggregates. The amyloid fibrils growth of α-syn occurs *via* a nucleation-dependent polymerization mediated by transient electrostatic interaction (Kumari et al., 2021). After the slow primary nucleation process, α-syn fibrils are elongated by addition of α-syn monomers. In the next step, secondary nucleation occurs whereby amyloid fibrils undergo fragmentation or catalyze the formation of new amyloids from monomers on its surface. This process accelerates generation of α-syn amyloid fibrils.

On the cellular level, the α-syn pathology in PD is not confined to the cell soma, but is also prominent in neurotic processes (Kahle, 2007). The majority of abnormally deposited α-syn occurs in neurotic processes, usually at the presynaptic terminals, which has been suggested to evoke a severe pathological impact on synaptic function. In a study by Kramer and Schulz-Schaeffer (2007), a significant synaptic pathology with almost complete loss of dendritic spines at the postsynaptic area was observed. Numerous studies identified synaptic effects as key determinants of α-syn-induced neurotoxicity in cell culture and *in vivo* models based on overexpression of α-syn. Effects included loss of presynaptic proteins, decreased release of neurotransmitter, redistribution of SNARE proteins, enlargement of synaptic vesicles, and inhibition of synaptic vesicle recycling. Such effects are causal in synaptic and neurotic degeneration. However, the exact sequence of synaptic events and the exact timepoint in the cascade during which α-syn assumes its neurotoxic potential are yet to be determined. On the organ level, α-syn pathology is widespread in various brain regions in PD patients. According to Braak et al. (2006), on the staging of PD pathology, α-syn pathology typically starts at olfactory bulb and the dorsal vagal nucleus, gradually spreading to associative cortical regions at later stages. Abnormal α-syn deposition can occur early in PD (Braak et al., 2006). Toxic α-syn species, especially oligomers, are linked to autophagy/lysosomal dysregulation, synaptic dysfunction, mitochondrial disruption, endoplasmic reticulum stress, oxidative stress, and neuroinflammation, which can eventually initiate neurodegeneration and neuronal cell death (Gao et al., 2011; Colla et al., 2012; Guo et al., 2013; Mazzulli et al., 2016). Certain mutant α-syn oligomer species are known to aggregate faster than the wild-type α-syn, which may enhance their toxicity (Li et al., 2019), implying that the cytotoxicity and pathology of α-syn are determined by its distinct aggregation pathways and kinetics.

To date, there is still a lack of effective diagnostic techniques and disease-modifying treatments for PD. The current clinical standard for PD diagnosis is predominantly based on clinical history and the presence of motor symptoms, which are often accompanied by a risk of misdiagnosis and disease detection solely at advanced stages (Good et al., 2011; Marsili et al., 2018). Dopaminergic network dysfunction in PD patients may occur before onset of obvious behavioral and motor symptoms, thus, by the time motor symptoms surface, a significant amount of dopaminergic neurons would have been degenerated and the disease would have progressed to its advanced stages (Madeo et al., 2012; Kordower et al., 2013). Hence, a rapid and accurate screening or early diagnostic technique to detect PD at early stages and track disease progression will greatly aid in the treatment and prevention of PD. Moreover, there is also a paucity of reliable biomarkers to track PD progression, which is therefore difficult to predict PD prognosis and monitor effectiveness of treatment strategies. As for the current treatments for PD, they rely mainly on dopamine replacement therapy, dopaminergic drugs, and deep brain stimulation which are only capable of providing symptomatic relief and to delay disease progression, but do not work to tackle the issues at its root (Poewe et al., 2017). Thus, given the roles of α-syn in PD pathogenesis, α-syn oligomers and misfolded α-syn can potentially be used as biomarkers for early diagnosis for and therapeutic targets of PD (Fields et al., 2019). A combination of proper diagnosis, monitoring, and treatment for PD are essential to improve the quality of patient lives. However, developing diagnostic and therapeutic techniques that target α-syn can also be challenging due to the various conformational changes α-syn can adopt due to its environmental influences and interpatient variability.

**FIGURE 3:**
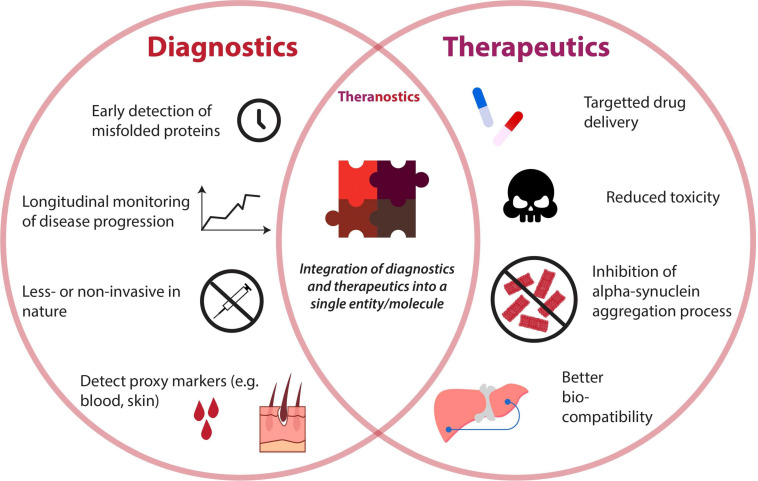
Venn diagram depicting attributes of diagnostics and therapeutics for detection and reduction of alpha-synuclein aggregates. A unison of both diagnostic and therapeutic capabilities gives rise to the booming field of “theranostics,” whose properties are also illustrated.

The emergence of nanotechnology and their rapid advancement in the last decade has offered revolutionary strategies for the diagnoses and therapies of various diseases (Azzawi et al., 2016; Padmanabhan et al., 2020). Nanoparticles (NPs) are defined as a natural or manufactured material comprising unbound or aggregated particles within the nano-size range of 1–100 nm (Soares et al., 2018). There are several types of NPs including natural inorganic particles, natural polymers, polyethylenimine derivatives, dendrimers, carbon-based nanoparticles, liposomes, micelles, solid lipid nanoparticles (SLNPs), and nanoemulsions (Pérez-Martínez et al., 2012; Naqvi et al., 2020). NPs can be functionalized, using appropriate molecules such as peptides, antibodies, and aptamers, to target the cellular elements or biomolecular pathway of interest to exert the desired effect (Sadat et al., 2016). Additionally, due to their size and relatively large surface area, NPs can also be an economic platform for the rapid development of innovation (Xia et al., 2016). Thus, NPs are ideal for developing new early diagnostic techniques and therapeutic agents for PD. The attributes of NPs applied as diagnostics, therapeutics, and theranostics are illustrated in Figure 3. This review will explore the current innovations in nanotechnology that target the α-syn aggregation pathway and how they can potentially be applied as effective early diagnostic and therapeutic solutions for PD.

## Early Diagnostic Nanotechnology Targeting α-syn

The challenges of PD diagnosis consist of the lack of a reliable biomarker and inexpensive tools for disease detection and disease monitoring. However, with the recent understanding of α-syn and development of new α-syn detection tools, recent research provides unprecedented insights into detection of α-syn in the cerebrospinal fluid (CSF), blood, and skin (Mollenhauer et al., 2018; Kim et al., 2019; Chang et al., 2020). Although α-syn was known to be an intracellular protein that aggregates and accumulates within neurons, there is compelling evidence to suggest that it may also be secreted by non-classical, ER/Golgi-independent exocytosis pathway or calcium-dependent exosomal release mechanism (Lee et al., 2005; Emmanouilidou et al., 2010). It was implied that these secretion mechanisms aid in inter-neuronal transmission of toxic α-syn oligomeric species and the propagation PD-related pathology (Desplats et al., 2009; Marques and Outeiro, 2012). Thus, developing a highly sensitive tool that can detect and monitor secreted α-syn in biological samples would be ideal for the non-invasive diagnosis and disease monitoring of PD.

Recent studies have focused on the development of new biosensors for αSyn detection. Biosensors are highly sensitive devices that can rapidly detect the concentration of specific biomolecules and, through the use of a transducer, are able to convert it into a measurable parameter (Metkar and Girigoswami, 2019). They are cost-effective tools that can be used for quick disease detection and progression monitoring to aid the planning of treatment. They are usually inexpensive, user-friendly and can be mass produced. Gold NPs are popularly used as a signal transducers in biosensors due to their localized surface plasmonic properties, which can help improve the detection sensitivity (Aldewachi et al., 2018; Jiang et al., 2018). They can be modified easily for various applications. Many recent studies have incorporated gold NPs to develop new biosensors that can detect α-syn.

You et al. (2019) designed an interdigitated electrode (IDE) biosensor in which a Gold NP-conjugated aptamer was used as a probe for the detection of α-syn on an Amine-Modified Dielectric Surface. The gold NP-conjugated aptamer was able to specifically recognize and detect α-syn with a detection limit of 10 pM (You et al., 2019). However, as this experiment was not performed on biological samples, the results could not be correlated with PD severity. Further testing and modification will be required to determine if this device is suitable for clinical diagnostic purposes. In another study, Karaboǧa et al. (2016) designed a gold nanoparticle (AuNP)–polyglutamic acid (PGA)-modified indium tin oxide (ITO)-based disposable neuro-biosensor system for the detection of α-syn in human CSF samples. The biosensor was able to accurately detect the α-syn levels in CSF with a detection limit of 0.135 pg/mL. The ability to detect α-syn in CSF is a good approach as molecular changes in CSF reflect the extracellular environment in the central nervous system (CNS). With further testing, this biosensor can potentially be applied for clinical diagnosis of PD *via* CSF.

Although the capacities of detecting α-syn are useful, the detection of toxic α-syn oligomers or toxic αSyn variants may be more indicative biomarker for PD. There are recent studies that have designed a more advanced biosensor that can detect the pathological form of α-syn. Tao et al. (2020) created an ultrasensitive poly (D-glucosamine)/gold nanoparticles/multi-walled carbon nanotubes/reduced graphene oxide (PDG/AuNPs/MWCNTs/rGO) modified immunosensor which can be used for the selective detection of α-syn oligomer in human plasma samples. Since α-syn oligomers are known to be the toxic species that induce PD pathology, detection of α-syn oligomers in human plasma can be useful in PD diagnosis. In another study, Khatri et al. (2018) designed a compact, label-free, chitosan-nanogold matrix based fiber-optic sensor which can be used to detect and differentiate monomeric from fibrillar α-syn with high specificity. As inhibition and breaking down of α-syn aggregates are ideal approaches in the treatment of PD, this biosensor can potentially be useful in studying and monitoring the effect of therapy in ameliorating Lewy body formation. Yin et al. (2020) designed a highly sensitive and specific surface plasmon resonance (SPR)-based immunosensor for quantitative evaluation of total α-syn and phosphorylated α-syn levels in CSF. In this device, anti-α-syn antibody (anti-αS) immobilized gold chips were used to capture α-syn, and Ti4+@TiP nanoparticles were added later to differentiate the phosphorylated α-syn (Yin et al., 2020). This device simplifies the process of quantifying total α-syn and phosphorylated α-syn, which make it more convenient, economical, and practical for clinical diagnostic uses. The ability to quantify phosphorylated α-syn is essential since phosphorylated α-syn is the major pathological variant associated with aggregation and neuroinflammation (Karampetsou et al., 2017).

Overall, all these biosensors mentioned were demonstrated to be rapid, highly specific, sensitive, stable, and reproducible, which make them a potential platform for clinical diagnostic. Once these biosensors are developed further and passed the necessary phases of clinical trials, they can potentially be used as effective non-invasive analytical tools for the screening, early diagnosis, and disease monitoring of PD.

## Therapeutic Nanotechnology Targeting α-syn Aggregation

Nanotechnology can provide many advantages to modern medicine. This includes highly specific targeted drug delivery, better biocompatibility, increased therapeutic efficacy, reduced toxicity, multi-functionalization, long circulation time, and controlled drug release at the targeted site (Choi and Han, 2018; Padmanabhan et al., 2020). Possible approaches to treating PD can be through inhibition of α-syn expression, inhibition of aggregation process, immunotherapy, and the promotion of α-syn degradation. Table 1 lists the different nanocarrier-mediated strategies of targeting PD.

**TABLE 1:** List of common nanocarriers used to transport therapeutic agents to biological targets implicated in PD.

References	Nanocarrier	Delivery approach	Therapeutic delivery molecule	Biomolecular target	Effect(s)	Model
Piersimoni et al., 2020	Gold-NPs	-	Lipoic acid	α-syn	- Inhibition of ROS formation - Reversal of cellular damage	*In vitro* cell model
Zhao et al., 2020	Ferulic acid and tannic acid NPs	-	Ferulic acid and tannic acid	α-syn	- Inhibition of α-syn fibrillation - Suppression of inflammation and oxidative stress	*In vitro* cell model
Hu et al., 2018	Gold NPs functionalized with chitosan	RNAi therapy; intraperitoneal injection	Plasmid DNA (pDNA)	*SNCA* gene	- Inhibition of dopaminergic neuronal death - Downregulation of *SNCA* expression	*In vivo* mouse model
Helmschrodt et al., 2017	Polyethyleneimine NPs	Intracerebroventricular injection	Small interfering RNAs (siRNAS) against *SNCA*	*SNCA* gene	- Downregulation of *SNCA* expression	*In vivo* mouse model
Liu et al., 2020	Rabies virus glycoprotein (RVG) peptide-modified exosome curcumin/phenylboronic acid-poly(2-(dimethylamino) ethyl acrylate)	Intraperitoneal injection	siRNA and curcumin	α-syn	- Inhibition of α-syn aggregation - Reduction of ROS generation - Reduction of α-syn cytotoxicity in dopaminergic neurons	*In vivo* PD mouse model
Asthana et al., 2020	Zinc oxide NPs	-	NPs themselves served therapeutic purpose	α-syn	- Interference with α-syn fibrillation - Flocculation of α-syn	*In vitro* cell model
Aliakbari et al., 2018	Zwitterionic nanoliposomes functionalized with PEG and cholesterol	-	NPs themselves served therapeutic purpose	α-syn	- Disruption of α-syn fibrillation - Reduction of ROS levels and neurotoxicity	*In vitro* cell model
Zand et al., 2019	Cerium oxide (CeO_2_) NPs	-	NPs themselves served therapeutic purpose	α-syn	- Inhibition of cytoplasmic α-syn foci accumulation - Reduction of cytoplasmic inclusion formation of α-syn - Reversal of α-syn-induced mitochondrial dysfunction	*In vitro* yeast model
Liu et al., 2019; Zha et al., 2016	Superparamagnetic iron oxide NPs (SPIONs)	Intravenous injection	Amyloid oligomer-specific scFv antibody	Amyloid oligomers (mutant Huntingtin and α-syn)	- Generation of MRI signal upon binding to amyloid oligomers - Interruption of amyloid aggregation by W20 - Improvement of motor and cognitive functions in mice	*In vivo* mouse model
Kim et al., 2018	Graphene quantum dots (GDQs)	Intraperitoneal injection	NPs themselves served therapeutic purpose	α-syn	- Inhibition of α-syn fibrillation - Disaggregation of existing mature fibrils - Amelioration of mitochondrial dysfunction - Rescue of dopaminergic neuron death and synaptic dysfunction	*In vivo* mouse model

*The therapeutic delivery molecules, mode of delivery of nanocarriers and expected effects are listed.*

A major challenge in designing a therapeutic strategy for the brain is the crossing of blood–brain barrier (BBB). The BBB is a semi-permeable membrane that selectively restricts the diffusion of foreign materials from the peripheral blood to the CNS (Daneman and Prat, 2015). Many drugs have shown promise in PD treatment but failed to cross the BBB. The inability to cross BBB prevents the drugs from reaching their target sites and an adequate accumulation of drugs at the sites to exert significant therapeutic effect. The development of nanocarriers may help in overcoming this challenge. Nanocarriers can be functionalized to cross the BBB, target desired cells, and allow a sustained release of therapeutics to specific brain targets (Zhou et al., 2018). Moreover, nanocarriers can protect encapsulated drugs from premature degradation to maximize the drug therapeutic efficacy. Hence, an increasing number of nanocarriers have been approved for clinical uses (Anselmo and Mitragotri, 2019). Several studies have successfully used nanocarriers to improve the delivery of PD therapeutic drugs and dopamine across BBB (Barcia et al., 2017; Wang et al., 2018; Jahansooz et al., 2020). The use of nanocarriers was reported to not only help improve the therapeutic efficacy, but also reduce drug side effects. Although these results are commendable, delivery of PD drugs and dopamine only work to relieve PD symptoms but do not resolve α-syn aggregation. Considering this, recent studies have turned to the employment of nanocarriers to deliver antioxidants that are capable of inhibiting α-syn fibrillation and reducing inflammation in the brain. Accumulation of toxic α-syn aggregates is known to trigger microglial activation and stimulate adaptive immunity to enable α-syn clearance, which could result in neuroinflammation and ultimately neuronal cell death (Tansey and Goldberg, 2010). Antioxidants are ideal therapeutic agents against PD neuroinflammation as they are biocompatible and able to repair oxidative stress-induced cell damage, but their low physiological concentration, short half-life and poor bioavailability can critically limit their therapeutic effect.

Recent studies have explored the use of nanocarriers to improve delivery of antioxidants to the brain. Lipoic acid is a powerful antioxidant that is naturally located in the mitochondria (Zhao et al., 2017). Piersimoni et al. (2020) demonstrated that gold NPs were used as a nanocarriers for lipoic acid (GNPs-LA). Gold NPs are ideal drug carriers due to their enhanced biocompatibility, stability, and ease of functionalization. The study demonstrated that the nanocarrier was able to successfully inhibit ROS formation and reverse cellular damage caused by excessive oxidative stress associated with α-syn aggregation in SH-SY5Y cells (Piersimoni et al., 2020). These results have shown that GNPs-LA may potentially be used as nanocarriers for antioxidant agents. Zhao et al. created an NP formulation that combined both ferulic acid and tannic acid (TA). TA is capable of inhibiting α-syn oligomerization and fibrilization (Caruana et al., 2011). Ferulic acid is effective as a radical scavenger and anti-inflammatory agent, which help suppress oxidative stress and inflammation (Srinivasan et al., 2007). The delivery of aggregation-inhibiting antioxidants was demonstrated to have a strong inhibitory effect on α-syn fibrilization by inhibiting α-syn internalization and intracellular α-syn oligomer formation in microglia. This was found to suppress the activation of a pro-inflammatory microglial phenotype and reduce the secretion of pro-inflammatory cytokines, which subsequently reduced ROS production and neuroinflammation (Zhao et al., 2020). Although these two studies obtained their results from *in vitro* cell models, they hold great promise in using antioxidant-encapsulated NPs in the future as a targeted anti-inflammation therapy for PD.

Nanocarriers can also be used to improve the delivery of gene therapy. RNA interference (RNAi) therapy can be used to selectively downregulate protein expression by silencing genes (Tseng et al., 2009). The challenge with RNAi therapy is that the naked siDNA possesses a short half-life and its negatively charged nature prevent it from diffusing across intracellular barrier (Endoh and Ohtsuki, 2009). Furthermore, endosomal capture or lysosomal degradation can hinder delivery. Thus, encapsulating siRNA in nanocarriers can provide them with stability, protect them from enzymatic degradation and facilitate their delivery to their target for sustained therapeutic effect. The surface of nanocarriers can be functionalized with molecules such as cell-penetrating peptides or PEG to enable endosomal escape and intracellular targeting (Parodi et al., 2015).

**FIGURE 4:**
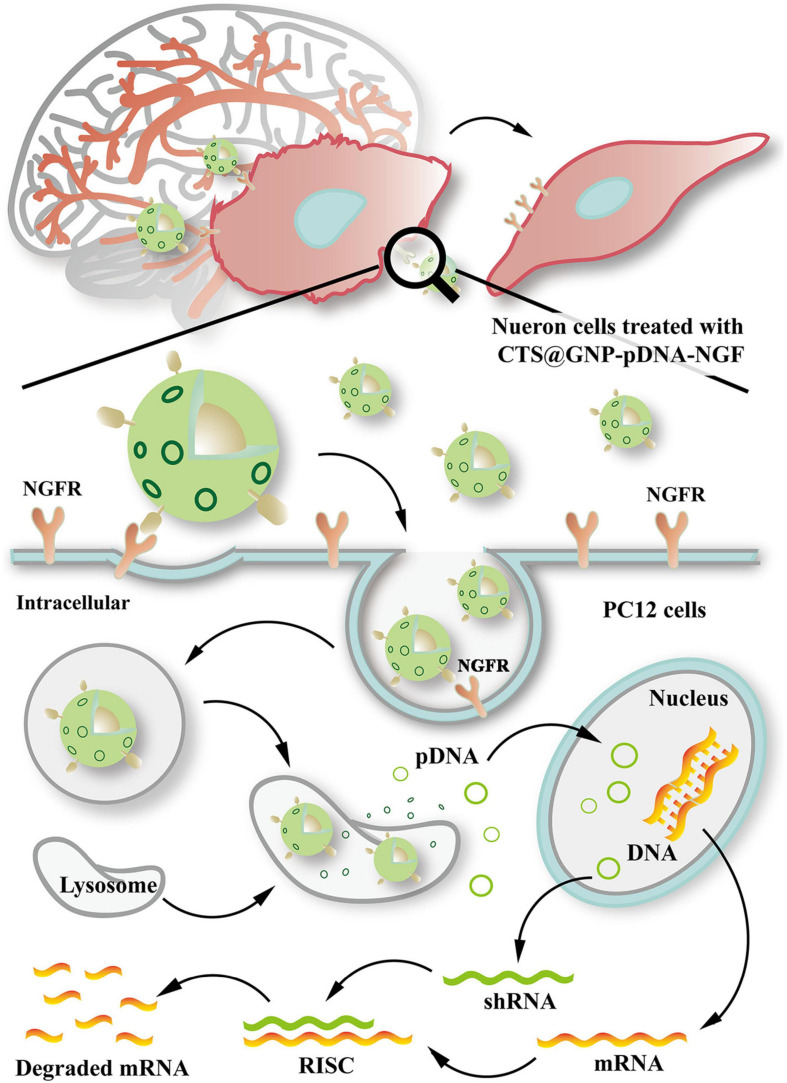
Schematic diagram illustrating the internalization of gold nanoparticle composites by PC12 cells and the molecular cascade leading to formation of RNA-interfering silencing complex (RISC), which suppresses SNCA expression *via* degradation of mRNA. Reprinted from Hu et al. (2018), with permission from Elsevier.

Several studies have shown the effective delivery of RNAi therapy *via* nanocarriers to suppress *SNCA* expression. Hu et al. (2018), through a mouse model experiment, designed gold NP composites (CTS@GNP-pDNA-NGF) to deliver plasmid DNA to the brain. Gold NPs were functionalized with chitosan, which help pDNA bind to the NP and protect it from degradation. The NPs were then further functionalized with nerve growth factor (NGF) to facilitate entry into dopaminergic neurons *via* NGF receptor-mediated endocytosis. It was demonstrated that the composite was not only able to successfully cross the BBB, but also inhibit apoptosis of the dopaminergic neurons in the PD mouse model by downregulating *SNCA* expression of α-syn. Figure 4 shows a schematic diagram expounding the molecular cascade that is set off upon the internalization of gold NPs by PC12 cells, eventually leading to a suppression of the SNCA gene (Hu et al., 2018). In another study, Helmschrodt et al. (2017) complexed polyethylenimine (PEI) NPs with synthetic non-coding small interfering RNAs (siRNAs) against *SNCA* to deliver RNAi therapy in PD mouse model. PEIs help protect siRNA from degradation by forming non-covalent complexes with the nucleic acids. It was demonstrated that a single administration of 0.75 μg of the siRNA-complexed NPs into CSF was sufficient to downregulate *SNCA* mRNA expression extensively across CNS in PD mouse model with no sign of adverse effect (Helmschrodt et al., 2017). Notably, another recent study designed a multifunctional NPs which can deliver both RNAi therapy and drug. Liu et al. (2020) designed an engineering core-shell hybrid system named rabies virus glycoprotein (RVG) peptide–modified exosome (EXO) curcumin/phenylboronic acid-poly[2-(dimethylamino)ethyl acrylate] nanoparticle/siRNA targeting SNCA (REXO-C/ANP/S), which can act as a nanoscavenger and SNCA inhibitor. Nanocomplexes coloaded with siRNA and curcumin were coated with REXO, exosomes modified with RVG peptide, which could specifically bind to the acetylcholine receptor and facilitate entry across BBB and neurons. Since curcumin is a neuroprotective drug known to inhibit α-syn aggregation, the combination of siRNA and curcumin work synergistically to down regulate α-syn expression, inhibit α-syn aggregation and reduce ROS production, which subsequently help to reduce the cytotoxicity of α-syn aggregates in dopaminergic neurons (Sharma and Nehru, 2018; Liu et al., 2020). All these studies illustrated the promising result of nano delivery of RNAi therapy in mouse model, thus further studies will be required to validate their uses in humans.

In addition to serving as a nanocarrier, NPs by themselves can also be used as a therapeutic agent. It was estimated that α-syn amyloid fibrils are formed by self-polymerization reactions that involve primary and secondary nucleation reactions and fibrils elongation (Linse, 2017). This is an electrostatically driven process which can be interfered by charged nanoobjects (Mohammad-Beigi et al., 2019). Several studies have exploited this property to inhibit α-syn aggregate formation. Asthana et al. (2020) discovered that the interaction between zinc oxide NPs with negative surface potential interface and α-syn can interfere with α-syn fibrillation and cause flocculation of α-syn, which significantly lower cytotoxicity in cell model experiment. This demonstrated that zinc oxide NPs may be used as a material for future therapeutics for PD. Similarly Aliakbari et al. (2018) reported the use of neutral (zwitterionic) nanoliposomes (NLPs), supplemented with cholesterol (NLP-Chol) and decorated with PEG (NLP-Chol-PEG) in disrupting α-syn fibrillization, which subsequently reduced ROS levels and neurotoxicity in cell model. In another study performed in α-syn-expressing yeast model, cerium oxide nanoparticles (CeO_2_ NPs) were discovered to be able to inhibit cytoplasmic α-syn foci accumulation and reduce formation of cytoplasmic inclusion of α-syn in the cell by interacting with α-syn. CeO_2_ NPs are known to have free radical scavenger properties (Pirmohamed et al., 2011; Dowding et al., 2012). Thus, CeO_2_ NPs were demonstrated in this study to decrease ROS production in neuronal cells, which help to reverse α-syn-induced mitochondrial dysfunction and increase the viability of α-syn-expressing cells (Ruotolo et al., 2020). This fit with another study which also demonstrated that CeO_2_ NPs can disrupt the formation of α-syn aggregates (Zand et al., 2019). Thus, these studies implied that CeO_2_ NPs may be used in the future as a nanomedicine for PD by acting as both a radical scavenger and as an inhibitor of α-syn toxicity in neuron.

## Promising Theranostics Nanotechnology Targeting α-syn Aggregation

Owing to the capacity of designing NPs to be multifunctional, they can also potentially be developed into theranostic entities that act as both disease monitoring and therapeutic agents. One of the NPs that can potentially be developed into a theranostics agent is superparamagnetic iron oxide nanoparticles (SPIONs). SPIONs are biodegradable and biocompatible MRI contrast agents that, due to their surface properties, can be engineered to penetrate the BBB and used as a nanocarrier for targeted drug delivery (Stephen et al., 2011; Wei et al., 2017). They can remain in circulation for a long time which can greatly enhance therapeutic effects of drug delivered. Their magnetic properties allow them to be tracked and enable a controlled drug release in real-time using an external magnetic field. Once magnetized, they can enhance drug deposition at the target site, which can contribute to improving the therapeutic efficacy of drug (Liu et al., 2013). MRI is a non-invasive imaging technique that has been used to detect underlying pathological processes and widely used for the diagnosis of various neurodegenerative diseases (Agosta et al., 2017). By integrating SPIONs into treatment regimen, multimodal molecular imaging can be performed with high sensitivity, resolution, and specificity.

**FIGURE 5:**
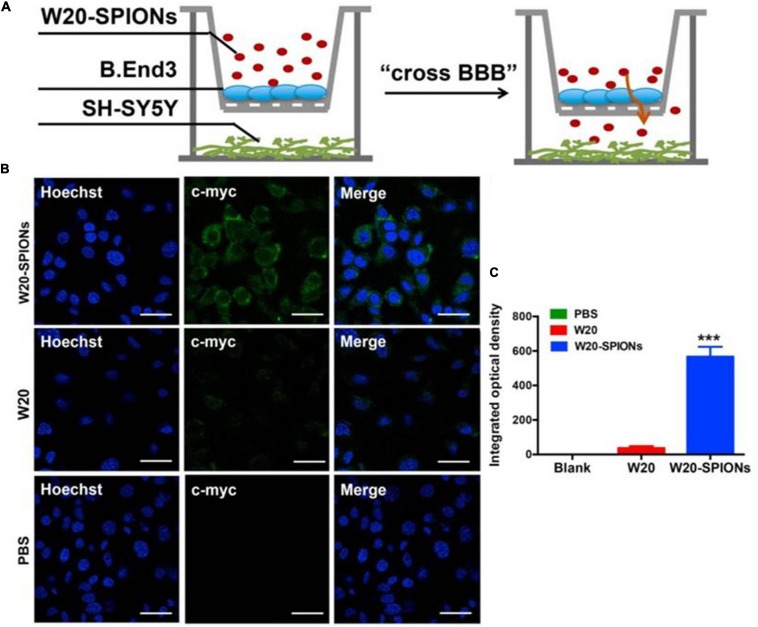
**(A)** The schematic illustration of *in vitro* BBB transwell system. **(B)** b. End 3 cells were incubated with W20-SPIONs, W20 or PBS, and their uptake in SH-SY5Y cells was detected by anti-c-myc antibody and subsequently with secondary antibody conjugated to Alexa Fluor 488. Scale bars, 20 μm. **(C)** Quantitative immunofluorescence analysis of W20 signal in SH-SY5Y cells. Reprinted from Liu et al. (2018), with permission from Elsevier. ***p < 0.001.

Recent studies have demonstrated the combined use of SPIONs and MRI to target alpha-synucleinopathies. For instance, Liu et al. (2018) conjugated an amyloid oligomer-specific scFv antibody (W20) to PEGylated SPIONs as a targeted MRI probe for detection of amyloid oligomer in PD mouse model. PEG-coating of SPIONs helped to reduce plasma protein binding, delayed clearance by the reticuloendothelial system and enhanced the particle circulation time, which allowed the SPIONs to be retained in the body for a longer duration (Qiao et al., 2012). It was demonstrated that the W20 conjugated PEGylated SPIONs were able to successfully cross the BBB and specifically bound to the oligomer to generate an MRI signal. Figure 5A illustrates a schematic of the BBB transwell system that was set up *in vitro.* As in Figures 5B, C, W20-conjugated SPIONs showed a heightened fluorescence intensity in neuroblastoma cells, indicative of them crossing the monolayer of b. End 3 cells (Liu et al., 2018). This can potentially be used for early-stage diagnosis for PD and for monitoring PD progression. Additionally, it was demonstrated in other studies that W20 can also interrupt amyloid aggregation to reduce oligomer-induced cytotoxicity and simultaneously improve motor and cognitive functions in PD mouse model (Zhang et al., 2011; Zha et al., 2016). Thus, these may suggest that W20-SPIONs, with further modification, could potentially be developed into theranostics agents that can be used to diagnose and monitor PD progression, and exert α-syn aggregation inhibitory effect to ameliorate PD.

Superparamagnetic iron oxide nanoparticles were also demonstrated to be an effective nanocarrier. Wang et al. (2018) designed a magnetic targeting nanocarrier Fe3O4-modified Res liposome (Res-lips@Fe3O4) to deliver resveratrol to brain tissues *via* intranasal delivery. Resveratrol is a polyphenol phytoalexin which possesses antioxidant and neuroprotective properties. The therapeutic efficiency of resveratrol is often limited due to its poor bioavailability in the CNS, unfavorable pharmacokinetic characteristics, and inability to cross BBB. This lipophilic nanocarrier is not only biocompatible and biodegradable but is also able to cross the BBB easily. Under the influence of external magnetic field, the nanocarrier can sustain slow release of resveratrol content at the target site in PD rat model, which helped to achieve superior therapeutic effect by reduce oxidative stress and inflammation (Wang et al., 2018). This illustrates that SPIONs may potentially be used to allow the monitoring of drug release in real-time, but also achieve a magnetically guided drug delivery to ameliorate alpha-synucleinopathies.

Superparamagnetic iron oxide nanoparticles can also be applied to gene therapy. Niu et al. (2017) created multifunctional SPIONs to deliver anti-αSyn shRNA plasmid into neurons by coating it with oleic acid molecules and N-isopropylacrylamide (NIPAm-AA), and functionalized it with NGF. NGF is used to specifically facilitate the neuronal uptake *via* NGF receptor-mediated endocytosis. The thermo-responsiveness and pH sensitivity characteristics of N-isopropylacrylamide (NIPAm-AA) allowed for a targeted and controlled release of ShRNA plasmid. It was reported to successfully inhibit programmed cell death in PD mouse model by downregulating α-syn expression, hence preventing the cytotoxic effects of α-syn in neurons (Niu et al., 2017). Thus, this may show promise of using SPIONs to perform gene therapy and monitoring PD progression.

Additionally, another study has shown that superparamagnetic iron oxide (SPIO)-gold (Au) nanomedicine functionalized with NGF can pose neuroregenerative effects. Yuan et al. (2018) designed an NGF functionalized SPIO-gold (Au) nanomedicine. NGF is essential for neuronal growth and differentiation (Aloe et al., 2016). However, due to its short half-life and inability to easily cross BBB, its therapeutic effect is limited. Thus, delivery of NGF across the BBB help to enhance the therapeutic efficiency of NGF in promoting neuronal growth. It was reported in the study that this nanomedicine can successfully stimulate neuron growth and differentiation under the influence of external magnetic fields and dynamic magnetic fields (Yuan et al., 2018). Thus, this may suggest that NGF-coated SPIONs can potentially be used for neuroregenerative therapy and simultaneously to monitor the neurodegenerative processes in PD.

Superparamagnetic iron oxide nanoparticles have also been illustrated for use in stem cell therapy as an MRI label for cell tracking (Asil et al., 2020). SPIONs were reported to have successfully delivered mesenchymal stem cells to the lesion site in the rat model of Huntington’s disease. They was also able to produce real-time and long lasting MRI signal for monitoring stem cell integration for at least 60 days (Moraes et al., 2012). Thus, the use of SPIONs as a nanocarrier for stem cells can potentially be applied to PD, which not only can enhance the therapeutic efficiency of stem cell therapy by facilitating delivery, but also help to monitor the therapeutic effect after delivery and improve the safety profile of the therapies.

However, it should also be noted that even through a low concentration of SPIONs can inhibit fibrillation, higher concentration of SPIONs can significantly speed up fibrillation processes (Ahmadianpour et al., 2019). As SPIONs are biodegradable, they are typically degraded by an intracellular lysosome-mediated degradation mechanism, which converts them to free iron that can be used by the body. However, high level of SPIONs will result in an accumulation of iron, which is associated with alteration in gene expression profiles, disturbance in iron homeostasis, oxidative stress, and altered cellular responses (Wahajuddin and Arora, 2012). Thus, the optimal concentration of SPIONs should be meticulously considered before they can be used on human subjects. There is also a need to ensure that SPIONs are eliminated from the body to prevent accumulated toxicity. To minimize intracellular catabolism and reactive oxygen species generation, renal excretion is considered the most desirable. To optimize the clearance rate through kidney, the size of SPIONs and the physiochemical properties of its coating materials on SPIONs must be carefully considered in its design.

**FIGURE 6:**
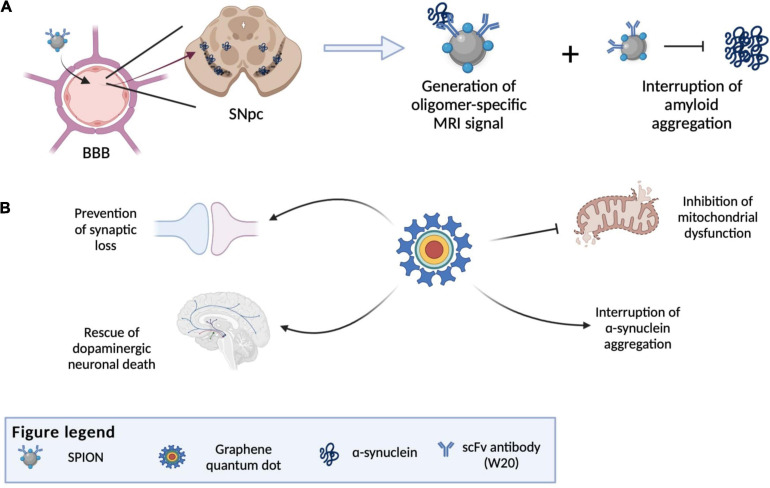
Illustration of various nanotheranostic approaches targeting different stages of PD. **(A)** Illustration of antibody-conjugated SPION bypassing BBB due to receptor-mediated transcytosis and finally reaching the substantia nigra. SPIONS are able to bind to amyloid and generate an MRI signal (diagnostic) and at the same time interrupt further aggregation of amyloid. **(B)** Examples of applications of graphene quantum dots (GQDs) in PD, whereby all mechanisms by which GQDs work give rise to an eventual rescue of neuronal death by interrupting α-synuclein aggregation, reducing reactive oxygen species generation and preventing synaptic loss. BBB, blood-brain-barrier; SNpc, substantia nigra pars compacta; SPION, superparamagnetic iron oxide.

Another notable NP that can potentially be used as a theranostics agent for PD is graphene quantum dots (GDQs). GDQs are graphene-based NPs popularly used in bioimaging as fluorophore due to their non-toxicity, excellent biocompatibility, effective renal clearance, photostability, extended fluorescence, inexpensive, and ability to cross the BBB (Younis et al., 2020). These properties also make GDQs increasingly optimal for developing targeted drug carriers and biosensing agents (Henna and Pramod, 2020). Recent studies have shown that GDQs themselves can also inhibit α-syn aggregation and promote disaggregation of mature fibrils (Mohammad-Beigi et al., 2019). This aggregation inhibitory effect of GDQs is dependent on its surface chemistry and dimensionality, which can interfere with the rate of protein aggregation, the mechanism of inhibition, and the morphology of the aggregated products (Ghaeidamini et al., 2020). The effect of GDQs was further illustrated by Kim et al. (2018). In their study, GQDs were shown to inhibit α-syn fibrilization and promote disaggregation of existing mature fibrils, which consequently ameliorate mitochondrial dysfunctions, rescue dopaminergic neuronal death and synaptic loss, and prevent behavioral deficit (Kim et al., 2018). Combining this α-syn aggregate inhibitory property of GDQs with their capabilities to be used as a drug carrier and bioimaging agent, further development of GQDs can innovate them into a theranostics agents for PD. However, it should also be noted that GDQs may promote the aggregation of certain variants of mutant α-syn in cells, which may contribute to the PD etiology (Mohammadi et al., 2017). Thus, the toxicity of GDQs demand carefully consideration before they can be utilized clinically. Figure 6 illustrates the various nanotheranostic approaches targeting different stages of PD.

## Future Perspectives

Despite the rapid advancements in the field of science, the definitive cause of idiopathic PD and mechanism for α-syn aggregation is still not well understood. Thus, development of new diagnostic methods that can monitor α-syn oligomerization could potentially improve the understanding of how PD arises and aid in finding new treatment strategies. At present, the most common way of tackling PD lies in providing symptomatic relief by the administration of dopamine precursors such as levodopa. In a nano-based approach of targeting PD, key molecular biomarkers such as the misfolded α-syn protein and genes such as *SNCA*, may be specifically targeted. An issue with direct dopamine administration to PD patients is the reduced bioavailability due to the molecule’s enhanced hydrophilicity. In this light, nanotherapeutics and theranostics take advantage of the carriers’ ability to be surface-modified to possess lipophilic characteristics and encapsulate hydrophobic drugs.

Notwithstanding the various innovations made in aiding α-syn detection, it should be noted that isolating optimal biological samples for PD diagnosis can still pose a challenge (Atik et al., 2016). Although CSF is ideal for the detection of brain-derived biomarkers as its molecular changes mimic the environment within the CNS, the surgical procedure to collect CSF can be too invasive, which may make it inconvenient for mass screening. Since α-syn from the CSF are readily transported into blood, blood is ideal for the mass screening of PD as it can be readily obtained less invasively. However, red blood cell contamination can elevate plasma α-syn and contribute to conflicting results which may interfere with accurate diagnosis (Barbour et al., 2008). A potential solution will be to design a diagnostic technique that would specifically detect CSF-derived α-syn or a pathological variant of α-syn such as oligomeric α-syn or phosphorylated-α-syn. Another option would be to detect α-syn in combination with another PD-related biomarker, such as Parkin, DJ-1, and PINK1 (He et al., 2018). Although α-syn can also be detected in skin and saliva, their correlation with the presence and severity of PD remains controversial. Further studies will be required to develop and validate suitable methods to accurately detect α-syn in the skin and saliva for PD diagnosis. Hence, future studies should focus on improving α-syn detection method in the CSF and blood for use in the clinical diagnosis of PD.

Nanotechnology brings about the possibilities of multifunctional therapeutic agents that can be used for diagnosis and treatment of PD, but there still exist underlying risks when translating them for clinical use. Although over the years an increasing number of NPs is being approved by the Food Drug Administration (FDA) for clinical purposes, nanomedicine is faced with multiple challenges in translation to medical applications due to lack of regulation and concern for nanotoxicity on the human body (Azhdarzadeh et al., 2015; Anselmo and Mitragotri, 2019; Foulkes et al., 2020). Despite the increasing use of nanomaterials in clinical settings over the past 10 years, there still exists a lack of consistent international definition and regulatory guidance for nanomaterials, which affects public acceptance of nano-products. Additionally, the potential toxicity of the NPs may also pose challenges for its translation to clinical uses. These challenges are even more profound in the development of NPs for use in neurodegenerative diseases due to the delicacy of the brain. Currently, there are still no NPs targeting α-syn or used to treat PD that are undergoing clinical trials. The studies mentioned in this review have been performed in cellular models and Parkinsonian animal models, and albeit the promising treatment methods, their toxicity in humans is still unknown and would have to be further evaluated. It was noted that the administration of certain NPs, such as titanium Dioxide NPs and zero-valent iron NPs, may worsen PD by enhancing α-syn fibril formation (Mohammadi and Nikkhah, 2017; Gilan et al., 2019). Thus, the properties of the NPs, their potential toxicity, and the optimal dosage necessitate careful consideration and modification accordingly before they can be applied in humans. Strategies must be put in place to lower the neurotoxicity induced by NPs. This can include removing toxic materials from NP composites, decrease the duration of NP exposure and coating the surface of NPs with molecules that can neutralize their neurotoxic properties. Thus, it is essential to develop standardized toxicological studies to examine the toxic effects and long-term impacts of NPs before their implemented use in human (Teleanu et al., 2018, 2019). Despite the potential toxicity, there is still optimism in the development of NPs for application in brain diseases. SGT-53 (SynerGene Therapeutics) is a cationic liposome with anti-transferrin receptor antibody, encapsulating wild-type p53 sequence used for the treatment of recurrent glioblastoma that is currently in phase II of clinical trials. It was found to be well tolerated and demonstrated enhanced antitumor activity in several patients (Pirollo et al., 2016). Cornell dots, a silica nanoparticle with a NIR fluorophore, PEG coating, and a 124I radiolabeled cRGDY targeting peptide designed for imaging of melanoma and malignant brain tumors are also undergoing clinical trials (Anselmo and Mitragotri, 2019). Another recent clinical trial by virtue of gadolinium-based NPs was supported in stereotactic brain directed radiation for metastatic incidences, demonstrating BBB traversal (Cagney et al., 2019).

Another consideration is the route of administration, metabolism, and elimination. Intracranial administration is considered the most effective method to deliver therapeutic NPs for brain as it can bypass the BBB. However, such invasive surgical method is too risky for use in clinical settings. Thus, the current trend in the development of therapeutic NPs is administration *via* intravenous route as it is considered to be the safer alternative and has shown promising results in various studies (Conceição et al., 2016). However, the effectiveness of therapy will be dependent on the ability of the NPs to cross BBB. As mentioned earlier in the review, NPs have been shown *via in vivo* mouse model studies to traverse the BBB when functionalized with specific molecular tags such as antibodies that would be able to exploit the receptor-mediated transcytosis pathways across the BBB. Notably, increasing studies have also demonstrated the use of intranasal delivery of NPs for brain-related disease (Sood et al., 2014; Battaglia et al., 2018). Intranasal delivery is a non-invasive method, which allow NPs to bypass the systemic circulation and allow more targeted delivery of NPs to brain and CSF through the intra- and extra- neuronal pathways. However, NPs can still be prone to enzymatic degradation and poor nasal mucosal barrier permeability may ensue. Therefore, further studies will be needed to improve and validate therapeutic delivery *via* intranasal route.

When designing therapeutic NPs for use in human, it is not only important to prevent premature elimination or degradation, it also important to consider how they can be metabolized and eliminated after their objectives are achieved so as to not cause accumulation of NPs in body and unintended adverse effects (Teleanu et al., 2018, 2019; Saeedi et al., 2019). NPs need to be well characterized at the physiochemical and physiological/biological levels to predict how the NPs will interact in a biological system and be eliminated from the body. There are two main clearance pathways for NPs: (1) by urinary excretion or (2) by hepatobiliary and feces excretion (Wei et al., 2018). NPs that are small (<5.5 nm) are quickly eliminated by kidney filtration and excreted in urine. NPs eliminated *via* this route typically only last in the body for few hours, which could affect them from attaining desired therapeutic efficiency. Most NPs that are large (>5.5 nm) and non-biodegradable are taken up and metabolized by the liver and commonly excreted *via* hepatobiliary elimination into feces. When eliminated *via* this route, NPs must be sequestered and chemically or physically processed by liver non-parenchymal cells (e.g., Kupffer cells and liver sinusoidal endothelial cells), which is a slow process that could result in accumulation of NPs in the liver and cause hepatotoxicity. Overcoming liver accumulation is a major issue to consider when designing intravenously administered nanomedicine. NPs that need to be eliminated *via* the liver can persist in the body for months, which could cause toxicity (Poon et al., 2019). The studies presented in this review are largely focused on optimizing the therapeutic effects of their NPs but lack data on the subsequent metabolism and elimination out of the body. Thus, future research should carefully consider metabolism and elimination profiles while designing more biodegradable and biocompatible NPs that can be safely used in humans.

As illustrated in our review, nanotechnology has the potential to develop theranostic agents that act as both disease-monitoring and therapeutic agents. This is not only useful in studying disease progression and effectiveness of therapy but will also reduce the economic and social burden of PD patients. Thus, further development of nanotechnology should not only focus on developing nanocarriers, but also on developing theranostic agents.

## Author Contributions

FLZ contributed to the literature search, review, and manuscript writing. MP conceived the figures and contributed to the writing and editing of the manuscript. PP and BG contributed to the interpretation of the figures and editing of the manuscript. All authors read and approved the final manuscript.

## Conflict of Interest

The authors declare that the research was conducted in the absence of any commercial or financial relationships that could be construed as a potential conflict of interest.

## Publisher’s Note

All claims expressed in this article are solely those of the authors and do not necessarily represent those of their affiliated organizations, or those of the publisher, the editors and the reviewers. Any product that may be evaluated in this article, or claim that may be made by its manufacturer, is not guaranteed or endorsed by the publisher.
